# Molecular Evidence of Human T‑Cell Leukemia Virus Type 1 Transmission by Needlestick Injury in Healthcare Workers

**DOI:** 10.1002/jmv.71143

**Published:** 2026-09-10

**Authors:** Masahito Tokunaga, Madoka Kuramitsu, Masumichi Saito, Miyuki Yoshimori, Jun Odawara, Yoshikiyo Ito, Hitomi Nakamura, Yasuko Sagara, Mieko Ishii, Naomi Nojiri, Hazuka Yoshida‐Furihata, Wakako Kuribayashi, Takuo Mizukami, Kiyonori Miura, Atae Utsunomiya

**Affiliations:** ^1^ Department of Hematology Imamura General Hospital Kagoshima Japan; ^2^ Center for Next‐Generation Biologics Research, National Institute of Infectious Diseases Japan Institute for Health Security Tokyo Japan; ^3^ Department of Diagnostic Testing and Technology Research National Institute of Infectious Diseases, Japan Institute for Health Security Tokyo Japan; ^4^ Department of Virology II National Institute of Infectious Diseases, Japan Institute for Health Security Tokyo Japan; ^5^ Department of Rare Diseases Research Institute of Medical Science, St. Marianna University School of Medicine Kanagawa Japan; ^6^ Department of Nursing Management Imamura General Hospital Kagoshima Japan; ^7^ Department of Quality Japanese Red Cross Kyushu Block Blood Center Fukuoka Japan; ^8^ Department of Obstetrics and Gynecology Nagasaki University Graduate School of Biomedical Sciences Nagasaki Japan

**Keywords:** de novo infection, healthcare workers, HTLV‐1, needlestick injury, occupational exposure, transmission route

## Abstract

Human T‑cell leukemia virus type 1 (HTLV‑1) is a retrovirus that spreads primarily through cell‐to‐cell transmission. Occupational transmission of HTLV‐1 by needlestick injury is considered exceedingly rare, with no reported molecularly confirmed cases. We report two healthcare workers who experienced accidental needlestick injuries while caring for patients seropositive for HTLV‑1 and subsequently seroconverted. Full‑length HTLV‑1 proviral sequencing revealed complete nucleotide identity between source patients and infected nurses. These strains belonged to the HTLV‐1 subtype 1a Japanese subgroup but were distinct from 315 previously registered strains. Proviral integration site analysis demonstrated nonoverlapping integration profiles between source patients and healthcare workers, providing direct evidence of de novo HTLV‑1 infection rather than expansion of transferred infected cells. Longitudinal analysis in one case showed dynamic clonal turnover between 5 and 14 months after transmission; > 90% of infected‐cell clones were replaced, although the proviral load remained stable and low. Serological profiling revealed transiently elevated immunoglobulin M responses to Gag p19 peptides during early infection, followed by gradual immunoglobulin G maturation. These findings provide the first molecular confirmation of HTLV‑1 transmission via needlestick injury resulting in de novo infection, revealing an under‐recognized occupational exposure route and supporting reconsideration of post‐exposure testing strategies, particularly in endemic regions.

**Abbreviations**
ATL: adult T‐cell leukemia‐lymphoma
CDC: Centers for Disease Control and Prevention
CI: confidence interval
DDBJ: DNA Data Bank of Japan
HAM/TSP: HTLV‐1–associated myelopathy/tropical spastic paraparesis
HBV: hepatitis B virus
HCV: hepatitis C virus
HIV: human immunodeficiency virus
HTLV‐1: human T‐cell leukemia virus type 1
IgG: immunoglobulin G
IgM: immunoglobulin M
IS: integration site
LIA: line immunoassay
MFI: median fluorescent intensity
NCBI: National Center for Biotechnology Information
PCR: polymerase chain reaction
PVL: proviral load
RAISING: Rapid Amplification of Integration Sites without Interference by Genomic DNA contamination
WHO: World Health Organization

## 1 Introduction

Human T‐cell leukemia virus type 1 (HTLV‑1) infects an estimated 5–10 million people worldwide and remains highly endemic in Japan, the Caribbean, South America, Austro‐Melanesia, and parts of Africa [1]. While most infected individuals remain asymptomatic, approximately 5% develop severe diseases, including adult T‐cell leukemia‐lymphoma (ATL) and HTLV‑1–associated myelopathy/tropical spastic paraparesis (HAM/TSP), making the prevention of new infections a major public health priority [2, 3, 4, 5]. HTLV‑1 is generally transmitted via four major routes: mother‐to‐child transmission via breastfeeding, sexual contact, organ transplantation, and transfusion of HTLV‐1‐contaminated blood products [6, 7, 8].

Among these transmission routes, infection via breastfeeding and transfusion has been substantially reduced through population‑level interventions, including nationwide antenatal screening programs, promotion of formula feeding, and blood‑donor screening [9, 10]. These successes demonstrate that targeted preventive measures can markedly reduce specific transmission routes.

Needlestick injuries are a major occupational hazard in healthcare settings and well‐recognized routes of transmission for bloodborne pathogens such as hepatitis B virus (HBV), hepatitis C virus (HCV), and human immunodeficiency virus (HIV). The estimated risks of seroconversion following percutaneous exposure range from 6% to 30% for HBV in nonimmune individuals, approximately 1.8% for HCV, and approximately 0.3% for HIV [11]. The Centers for Disease Control and Prevention (CDC) has estimated that 385 000 needlesticks and other sharps‐related injuries are sustained by hospital‐based healthcare personnel annually in the United States [12, 13].

However, despite the well‐established role of needlestick injuries as an occupational transmission route for these major bloodborne viruses, occupational transmission of HTLV‐1 via needlestick injury has never been conclusively demonstrated. Consequently, needlestick injury remains a largely unrecognized potential route of HTLV‑1 transmission in clinical practice. Although transmission through needle sharing has been reported for HTLV‐2 among people who inject drugs [14], direct molecular evidence supporting HTLV‐1 transmission following occupational exposure is lacking. As a result, needlestick injury is not generally regarded as an important route of HTLV‐1 transmission, and the true occupational risk remains uncertain.

This report describes two nurses who experienced occupational needlestick injuries during clinical care of patients positive for HTLV‑1 and who subsequently seroconverted. Using full‐length proviral genome sequencing and integration‐site analyses, this report provides the first direct molecular confirmation of occupational needlestick injury‐mediated HTLV‐1 transmission and demonstrates that infection resulted from de novo viral transmission rather than transfer of infected cells. These findings identify an underrecognized occupational exposure route and have implications for post‐exposure testing strategies in HTLV‐1‐endemic regions.

## 2 Materials and Methods

### 2.1 Study Participants and Specimen Collection

This study included two healthcare workers who experienced occupational needlestick injuries while caring for patients with confirmed HTLV‐1 infection and subsequently underwent clinical evaluation for possible HTLV‐1 transmission in 2023–2024 and 2025, respectively. Blood samples were collected longitudinally from the exposed healthcare workers as part of post‐exposure follow‐up. Samples from the corresponding source patients were also obtained for molecular comparison. Plasma and peripheral blood specimens were used for serological, virological, and genomic analyses.

### 2.2 Plasma Processing and Genomic DNA Extraction

Blood samples were collected from the participants in ethylenediaminetetraacetic acid (EDTA)‐containing tubes, and plasma was separated by centrifugation. Genomic DNA was extracted from whole blood using a QIAamp DNA Blood Mini Kit (QIAGEN, Hilden, Germany) according to the manufacturer's instructions.

### 2.3 Antibody Test

Serological tests were performed using the INNO‐LIA HTLV‐I/II score (Fujirebio, Tokyo, Japan) according to the manufacturer's instructions. IgM was detected using INNO‐LIA HTLV‐I/II scores as previously described [15]. The antibody signal bands on the INNO‐LIA HTLV‐I/II score strip were obtained using an Odyssey M imaging system (LI‐COR Biotechnology, Nebraska, USA).

### 2.4 xMAP Immunoassay

N‐terminal biotinylated HTLV‐1 antigen peptides (Gag p19: Biotin‐PEG3‐RPAPPPPSSPTHDPPDSDPQIPPPYVEPTAPQVL, Env gp21: Biotin‐PEG3‐IVKNHKNLLKIAQYAAQNRRGLDLLFWEQGGLCKALQEQCCFLN) were synthesized by PEPTIDE Institute Inc. (Osaka, Japan). These regions correspond to previously reported immunodominant epitopes [16, 17].

MagPlex‐Avidin microsphere beads (Luminex, Tokyo, Japan) conjugated with HTLV‐1 antigen peptides were incubated with plasma samples using a Bio‐Plex Pro Human Serology Reagent kit (Bio‐Rad, CA) according to the manufacturer's instructions. Human IgG and IgM were detected using PE Mouse Anti‐Human IgG (clone G18‐145; BD Biosciences, NJ, USA; 1:6.5 dilution) and Horizon BV421 Mouse Anti‐Human IgM (clone G20‐127; BD Biosciences; 1:50 dilution), respectively. Fluorescence signals were analyzed using an INTELLIFLEX DR‐SE instrument (Thermo Fisher Scientific).

Specimens from asymptomatic carriers (*n* = 102) were randomly selected from the Joint Study on Predisposing Factors for ATL Development. Statistical analyses were performed using GraphPad Prism version 7 (GraphPad Software, San Diego, CA, USA).

### 2.5 Proviral Load Quantification

Proviral load (PVL) was quantified using the QIAcuity Digital PCR System (QIAGEN) according to the manufacturer's instructions. Briefly, genomic DNA was mixed with QIAcuity 4× Probe PCR Master Mix and HTLV‐1‐ and RPP30‐specific primer probes (sequences are listed in Supporting Information S1: Table 1 and as reported previously [18, 19]). The mixture was then loaded onto a QIAcuity 26k 24‐well nanoplate. Thermal cycling was performed according to the manufacturer's protocol. Quantitative results were obtained using the QIAcuity detection system and software (version 2.2.0.26).

### 2.6 Sequencing Analysis of HTLV‐1 Proviral Genomic Sequences

Full‐length HTLV‐1 genomic sequence was amplified from the three regions using a KOD One Polymerase Kit (Toyobo, Tokyo, Japan) according to the manufacturer's protocol. The primer sequences are listed in Supporting Information S1: Table 2. The resulting amplicons were purified using a QIAquick PCR Purification Kit (QIAGEN), and the sequences were read by Eurofins Genomics Inc. (Ebersberg, Germany) and ATENZA LIFE SCIENCE Inc. (South Plainfield, NJ, USA). All primer sequences used in the long PCR and sequencing PCR have been described previously [20]. Sequences were assembled using ATGC software (GENETYX, Tokyo, Japan). Complete long terminal repeat (LTR) sequences were determined by combining consensus regions of 5′‐LTR and 3′‐LTR reads, as described previously [21].

HTLV‐1 nucleotide sequences were submitted to the DNA Data Bank of Japan (DDBJ) under NCBI Accession Numbers LC815002 and LC924600.

### 2.7 Phylogenetic Analysis

For phylogenetic analysis, complete HTLV‐1 genomic sequences were aligned using MAFFT [22], and the alignment was curated with BMGE [23]. The phylogenetic tree was inferred using PhyML software. Node support was assessed using bootstrap analysis with 1000 replicates [24, 25].

The HTLV‐1 nucleotide sequences of Japanese asymptomatic carriers used in this study were as follows: LC209958–LC210071, LC859640–LC859841. AB513134 was used as a prototype of the HTLV‐1 complete sequence.

### 2.8 Proviral Integration Site Analysis of HTLV‐1‐infected Cells

The proviral integration sites of HTLV‐1–infected cells in peripheral blood were amplified using Rapid Amplification of Integration Sites without Interference by Genomic DNA contamination (RAISING) as previously described, and high‐throughput sequencing analyses were then performed by FASMAC Inc. (Kanagawa, Japan) [26]. Analyses were performed using Microsoft Excel (v16.109.3) and R (v4.5.2), with visualizations generated using the readxl and tidyverse packages.

### 2.9 Ethics Statement

This study was approved by the Ethical Review Board of the National Institute of Infectious Diseases (Institutional Review Board Approval No. 1331). The nurses and patients provided consent to participate in the study and for their clinical information to be shared in this report.

## 3 Results

### 3.1 Cases

Case 1 involved a 45‐year‐old female nurse who sustained an accidental needlestick injury to the left fourth finger with a 23‐gauge needle while caring for a patient with rheumatoid arthritis who was seropositive for HTLV‐1 on October 12, 2023. The wound was immediately squeezed out and washed under running water.

Case 2 involved a 57‐year‐old female nurse who sustained a deep finger puncture from a 16‐gauge needle contaminated with blood from a patient positive for HTLV‑1 antibodies during hemodialysis on June 27, 2025. The wound was immediately washed under running water and disinfected with ethanol.

In both cases, the source patient and exposed healthcare worker underwent post‐exposure antibody testing in accordance with institutional infection‐control protocols. HTLV‐1 antibody testing was negative in both nurses at the time of injury, but became positive 62 days (Case 1) and 76 days (Case 2) after exposure.

### 3.2 Genetic Concordance of Transmitted HTLV‑1 Proviral Sequences Between Individuals

**Figure 1:**
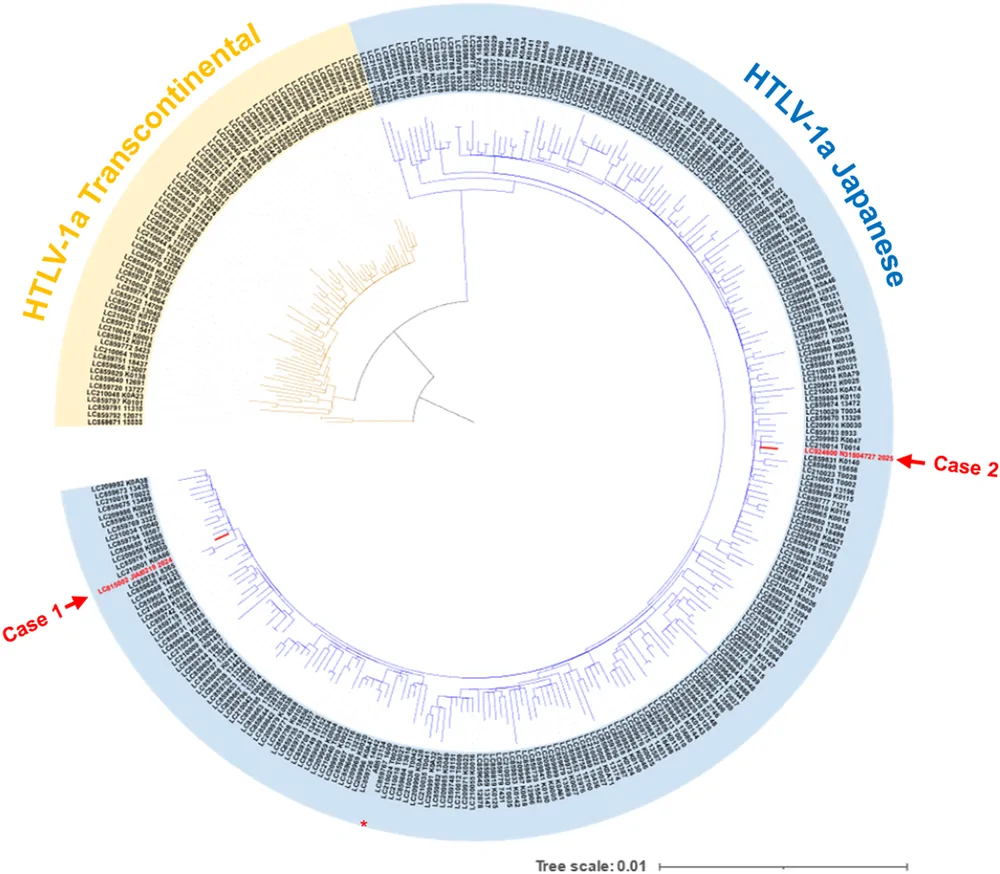
Phylogenetic analysis of HTLV‑1 strains transmitted by needlestick injury (NSI) in comparison with reference strains from Japanese asymptomatic carriers. Complete HTLV‐1 proviral genomic sequences were determined from the NSI source patients, together with 315 reference sequences registered in the NCBI database, including 314 strains derived from healthy Japanese blood donors that were previously determined by the authors, as well as a prototype strain (*: AB513134). The maximum‐likelihood phylogenetic tree was constructed using PhyML software. Red arrows: NSI‐transmitted strains. HTLV‐1, human T‐cell Leukemia virus type 1; NSI, needlestick injury.

First, to elucidate the transmission of HTLV‐1 from the patient to the nurse in both cases, full‐length proviral genomic sequences of the individuals were determined using Sanger sequencing. Full‐length proviral genomic sequencing demonstrated complete nucleotide identity between the source patient and nurse in both cases (Table 1). Additionally, phylogenetic analysis was performed by comparing the needlestick‐injury‐transmitted HTLV‑1 sequences with 315 reference strains previously registered in the NCBI database, including 314 complete proviral sequences derived from healthy Japanese blood donors determined by Sanger sequencing, as well as a representative prototype strain, AB513134. The transmission‐associated strains were unique and did not match any of the 315 reference strains (Figure 1).

**Table 1:** Clinical summary of cases of needlestick injury infection.

Case	Medical condition/occupation	Sex	Age (years)	Time after injury (months)	Proviral load (copies/100 cells)	Sequence homology	Provirus sequence accession number
*Case 1*							
Source	Patient with rheumatoid arthritis	Female	46	—	5.93	—	LC815002
Recipient	Nurse	Female	45	5	0.05	100% (9037/9037)^a^	LC815002
			46	10	0.06	—	—
			46	14	0.15	—	—
*Case 2*							
Source	Patient undergoing hemodialysis	Male	63	—	0.31	—	LC924600
Recipient	Nurse	Female	57	3	0.18	100% (9037/9037)	LC924600

^a^ Values in parentheses indicate the number of matched nucleotides divided by the total proviral sequence length of the corresponding patient.

These findings support the transmission of HTLV‐1 from the source patient to the nurse in both cases. Phylogenetic analysis revealed that these strains belong to the HTLV‐1a Japanese subgroup (Figure 1).

### 3.3 Evidence of HTLV‐1 De Novo Infection

Given the complete match in the proviral genome sequences, proviral integration sites were investigated using genomic DNA obtained from the peripheral blood of patients and nurses.

**Figure 2:**
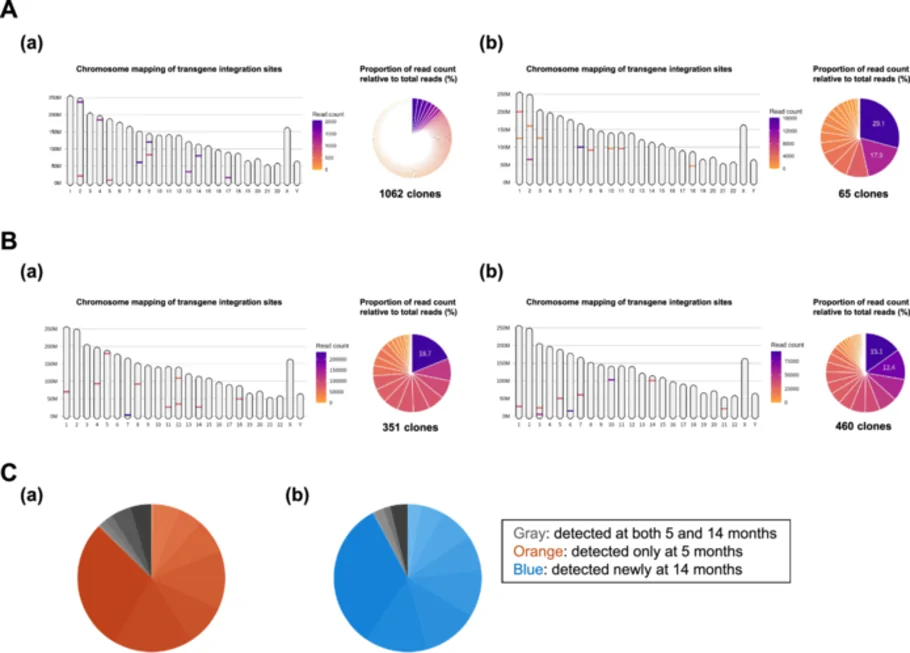
Transition of clonal proportions of HTLV‐1–infected cells analyzed by RAISING 3′LTR integration site analysis. (A) Proportion of clones observed in the patient (a) and nurse (b) in Case 1. (B) Proportion of clones observed in the patient (a) and nurse (b) in Case 2. Highlighted chromosomal positions indicate the top 10 HTLV‐1 integration sites. (C) Clonal dynamics of HTLV‑1–infected cells in Case 1. The paired pie charts show the relative abundance of HTLV‑1 proviral integration sites in Case 1 at 5 months (a) and 14 months (b). Integration sites detected at both points are shown in gray slices. Integration sites detected only at 5 months are shown in orange slices, whereas integration sites newly detected at 14 months are shown in blue slices. HTLV‐1, human T‐cell lymphotropic virus type 1; RAISING, Rapid Amplification of Integration Site without Interference by Genomic DNA contamination; LTR, long terminal repeat.

As shown in Figure 2, analysis of proviral integration sites showed 1062 and 65 clones, respectively, in Case 1, and 351 and 460 clones in Case 2. No identical integration sites were detected in either case, strongly suggesting de novo infections.

The diversity of HTLV‐1‐infected clones in nurses was further investigated in Case 1, at 5, 10, and 14 months postinfection. The dominant infected‐cell clones changed substantially over time, with most present only at a single time point (Figure 2C). At 10 months, the clones commonly observed at 5 months comprised only 18.3% of the total infected cells (Supporting Information S2: Figure 1). Several clones common at both 5 and 14 months were also identified but accounted for only 7.8% of all infected cells at 14 months. Most clones present at 5 months were no longer detectable at 14 months, and 92.2% of infected cells at 14 months emerged during the study period (Figure 2C). Despite these marked changes in clonal composition, PVL remained low and relatively stable throughout follow‐up (Table 1).

### 3.4 Early Humoral Immune Responses After HTLV‐1 Transmission

To characterize humoral immune responses during early HTLV‐1 infection following needlestick transmission, antibody profiles were examined using line immunoassay (LIA). In both nurses, LIA‐detected IgG revealed clear reactivity to all major HTLV‐1 antigens at all time points.

**Figure 3:**
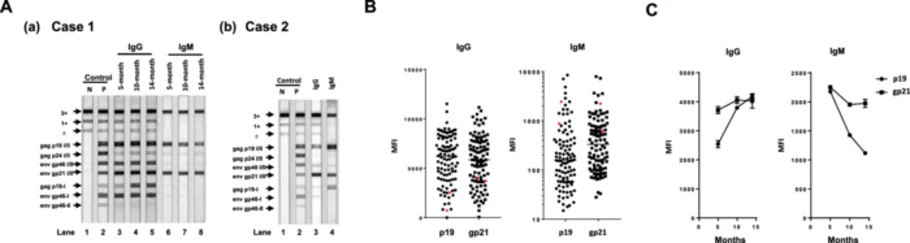
Transition of antibody titers for HTLV‐1 antigen in Case 1. (A) Line immunoassay with IgG (left) and IgM (right) at 5, 10, and 14 months after transmission in Case 1 and 3 months in Case 2. (B) IgG and IgM antibody titer of asymptomatic carriers for Gag p19 and Env gp21 peptide evaluated using xMAP magnetic bead‐based assay. Red dots with a diamond and a triangle indicate Cases 1 (5 months) and 2 (3 months) after HTLV‐1 infection, respectively. (C) Transition of IgG and IgM antibody titers for p19 and gp21 peptides in Case 1. HTLV‐1, human T‐cell Leukemia virus type 1; IgG, immunoglobulin G; IgM, immunoglobulin M; MFI: median fluorescent intensity.

Sequential samples in Case 1 demonstrated dynamic changes in antibody responses. Notably, the band intensity corresponding to gag p19‐I increased between 5 and 10 months postinfection (Figure 3A). Furthermore, human IgM showed strong reactivity to gag p19 and env gp21 antigens, especially at 5 months.

Based on the marked temporal changes observed in Case 1 for a subset of antigens, quantitative analysis of antibody titers was performed using an xMAP immunoassay with synthetic peptides corresponding to gag p19 and env gp21. Plasma IgG and IgM titers from Case 1 (at 5 months) and Case 2 were compared with those of asymptomatic carriers (*n* = 102).

At 5 months after infection, IgG responses against p19 and gp21 were lower than those observed in asymptomatic carriers, whereas IgM responses against both antigens were substantially higher (Figure 3B). IgG titers for p19 peptides gradually increased during the observation period, increasing by 1.65‐fold between 5 and 14 months, while IgM titers peaked at 5 months and gradually decreased by 0.51‐fold (Figure 3C). Changes in IgG and IgM titers against the p19 peptide correlated well with p19‐I band intensities in LIA. Taken together, these findings indicate progressive maturation of the antibody response during the early phase of HTLV‐1 infection.

### 3.5 Institutional Surveillance of Occupational HTLV‐1 Exposure

Institutional surveillance at Imamura General Hospital between 2011 and 2025 revealed 564 recorded incidents of needlestick or mucocutaneous exposure. Among these, 45 healthcare workers sustained needlestick injuries from patients positive for HTLV‑1. Of the 45 exposed healthcare workers, 2 were HTLV‐1 carriers and were already seropositive at baseline. No HTLV‐1 seroconversion was observed among the remaining 43 healthcare workers who were seronegative at the time of exposure (data not shown). In contrast, the two cases of seroconversion in this report were referred from outside institutions. These findings indicate that HTLV‐1 seroconversion following occupational needlestick exposure can occur, but was not observed among healthcare workers monitored through institutional surveillance during the study period.

## 4 Discussion

The World Health Organization (WHO) reports that sharps injuries cause an estimated 66 000 HBV, 16 000 HCV, and 2000–5000 HIV infections among healthcare workers worldwide each year [27]. These injuries have been implicated in the transmission of more than 20 pathogens, prompting the development of extensive preventive frameworks in infection control guidelines for these pathogens. However, comparable guidance for HTLV‑1 remains limited, largely because well‑documented occupational transmission events are rare, HTLV‑1–associated diseases develop only after prolonged latency, and most infected individuals remain asymptomatic throughout life. Collectively, these factors have contributed to the historically low recognition of occupational transmission risk. The present study provides the first direct molecular evidence of HTLV‐1 transmission following occupational needlestick injury. Complete nucleotide identity of the proviral genomes between source patients and healthcare workers, together with entirely distinct proviral integration profiles, demonstrated that infections resulted from de novo HTLV‐1 transmission events rather than expansion of transferred infected cells.

The present cases represent two contrasting exposure patterns: one characterized by a high PVL in the source patient with a superficial needlestick injury and the other by a low PVL with a deep percutaneous injury. How many infected cells are transferred during a needlestick injury remains unknown. Nevertheless, HTLV‑1 infection was established in both healthcare workers, indicating that transmission can occur under distinct exposure conditions. These observations suggest that both viral burden and injury characteristics influence transmission risk.

Previous studies of chronic HTLV‑1 infection have demonstrated remarkable long‐term clonal stability. In particular, Gillet and colleagues reported that once an infection is established, the majority of unique proviral integration sites persist for years, whereas newly detected clones are relatively uncommon [28]. Subsequent longitudinal studies have further shown that a subset of dominant HTLV‑1–infected T‑cell clones can be stably maintained over time [29].

In contrast, longitudinal analysis of Case 1 revealed substantial changes in clonal composition during the first 14 months after transmission. Most clones detected at 5 months were no longer observed at later time points, whereas numerous newly detected clones emerged during follow‐up. Despite these marked changes in clonal composition, PVL remained relatively stable at a low level. These findings suggest that early HTLV‐1 infection is characterized by dynamic clonal turnover before the establishment of the more stable clonal architecture observed during chronic infection. Continued longitudinal follow‐up is needed to determine when and how this stabilization occurs.

The clonal dynamics observed during early infection were accompanied by temporal changes in HTLV‐1‐specific antibody responses. IgM responses against Gag p19 and Env gp21 were highest during the early phase and declined over time, whereas IgG responses increased progressively during follow‐up. These findings are consistent with maturation of the humoral immune response following HTLV‐1 transmission and provide a unique view of immune responses during the early stages of infection.

Studies of horizontal HTLV‑1 transmission based on surveillance of seroconversion among repeat blood donors have estimated that approximately 4000 new horizontal infections occur annually in Japan [30, 31]. Most infections are thought to result from sexual transmission, with male‐to‐female transmission occurring approximately three times more frequently than female‐to‐male transmission.

Accumulating evidence indicates that HTLV‑1–associated diseases are not restricted to vertical transmission events. Development of disease following horizontal transmission has been clearly implicated in HTLV‑1–associated uveitis [32] and has also been linked to inflammatory and malignant complications, including HAM/TSP, particularly after hematopoietic and organ transplantation [33, 34], and adult T‑cell leukemia/lymphoma [35].

Because needlestick injury results in horizontal transmission of HTLV‐1, these observations suggest that occupationally acquired infection may also carry long‐term disease risks. Because a high HTLV‑1 PVL is a key risk factor for the development of HTLV‑1–associated diseases [36], continued longitudinal monitoring of PVL and related markers is warranted in individuals infected via needlestick injury.

The genomic stability of HTLV‑1 during horizontal transmission has previously been reported, notably for the *env* gene in married couples, in whom the *env* sequences were identical between spouses [37]. Consistent with these observations, complete nucleotide identity of the full‐length HTLV‐1 proviral genome was observed between the source patients and healthcare workers in both needlestick‐transmission cases examined in this study. Although the number of cases was limited, these findings further support the high genetic stability of HTLV‑1 during transmission.

Compared with other blood‐borne viruses, HTLV‐1 exhibits remarkably limited genetic diversity during transmission. HIV‑1 accumulates mutations at an approximate rate of 3 × 10^−5^ per base per replication cycle, corresponding to approximately one mutation per genome per cycle [38], whereas HCV rapidly establishes intra‐host sequence diversity shortly after infection [39]. HBV also gradually accumulates genetic diversity through error‑prone reverse transcription during chronic infection, with an estimated mutation rate of ~2 × 10^−4^ substitutions per site per year [40, 41]. In contrast, the complete genomic concordance observed in both source patient–healthcare worker pairs underscores the exceptional genetic stability of HTLV‐1. Taken together, these observations highlight that HTLV‑1 transmission can be traced at the molecular level with high precision, a feature that may facilitate transmission tracking and occupational exposure investigations.

This study has several limitations. First, because HTLV‑1 transmission via needlestick injury appears to be uncommon, the number of available cases was limited. In addition, blood samples could not be obtained during the earliest phase of infection before seroconversion. Consequently, detailed longitudinal data regarding early changes in PVL, clonal expansion, and antibody responses during the pre‐seroconversion period were unavailable. Second, serial blood samples collected between 5 and 14 months after infection were available for only one case, limiting the ability to determine whether the observed clonal and serological dynamics are representative of HTLV‐1 infection following occupational transmission. In addition, no samples were available from this healthcare worker beyond 14 months after infection, limiting assessment of the long‐term stability of the infected‐cell clone population. Third, because population‐based data on needlestick injury incidents outside our institute were unavailable, the true transmission risk associated with needlestick injury could not be estimated. Finally, long‑term clinical outcomes, including the future development of HTLV‑1–associated diseases, remain unknown. Continued follow‐up of affected individuals will therefore be important.

## 5 Conclusion

The results of this study provide the first molecular confirmation of HTLV‑1 transmission via needlestick injury and demonstrate that infection results from de novo viral transmission rather than transfer of infected cells. These findings identify an under‐recognized occupational exposure route and suggest that HTLV‑1 antibody testing should be incorporated into post‐exposure evaluations in Japan, especially in nonendemic areas. Moreover, HTLV‐1 antibody testing after needlestick injury should be reconsidered in clinical practice, regardless of endemicity, to improve occupational risk assessment and long‐term follow‐up. Finally, further studies are needed to define the incidence, long‐term clinical consequences, and optimal post‐exposure management of occupational HTLV‐1 transmission.

## Author Contributions

Conceptualization: Madoka Kuramitsu and Atae Utsunomiya. Data curation: Madoka Kuramitsu. Formal analysis: Madoka Kuramitsu and Masumichi Saito. Funding acquisition: Madoka Kuramitsu, Masumichi Saito, Takuo Mizukami, Kiyonori Miura, and Atae Utsunomiya. Investigation: Masahito Tokunaga, Madoka Kuramitsu, Masumichi Saito, Miyuki Yoshimori, Yoshikiyo Ito, Hitomi Nakamura, Yasuko Sagara, Mieko Ishii, Naomi Nojiri, Hazuka Yoshida, Wakako Kuribayashi, Takuo Mizukami, and Atae Utsunomiya. Methodology: Madoka Kuramitsu, Masumichi Saito, and Atae Utsunomiya. Project administration: Madoka Kuramitsu and Atae Utsunomiya. Resources: Masahito Tokunaga, Miyuki Yoshimori, Jun Odawara, Yoshikiyo Ito, Hitomi Nakamura, Yasuko Sagara, and Atae Utsunomiya. Supervision: Kiyonori Miura and Atae Utsunomiya. Validation: Madoka Kuramitsu. Visualization: Madoka Kuramitsu and Masumichi Saito. Writing – original draft: Masahito Tokunaga, Madoka Kuramitsu, Masumichi Saito, and Atae Utsunomiya. Writing – review and editing: Masahito Tokunaga, Madoka Kuramitsu, Masumichi Saito, Hitomi Nakamura, Yasuko Sagara, Takuo Mizukami, and Atae Utsunomiya. All authors have read and approved the final version of the manuscript.

## Ethics Statement

This study was approved by the Ethical Review Board of the National Institute of Infectious Diseases (Institutional Review Board Approval No. 1331).

## Consent

The nurses and patients provided consent to participate in the study and for their clinical information to be shared in this report.

## Conflicts of Interest

The authors declare no conflicts of interest.

## Supporting information


**Figure S1:** Clonal dynamics of HTLV‑1–infected cells in Case 1 at 5 and 10 months. HTLV‐1, human T‐cell Leukemia virus type 1.

**Table S1:** Genome‐specific primer probe sequences for HTLV‐1 proviral load dPCR. HTLV‐1, human T‐cell Leukemia virus type 1; dPCR, digital PCR. **Table S2:** Nested genomic long‐PCR primers for HTLV‐1 full‐length genomic sequencing. PCR, polymerase chain reaction; HTLV‐1, human T‐cell Leukemia virus type 1.

## Data Availability

The data that support the findings of this study are available on request from the corresponding author. The data are not publicly available due to privacy or ethical restrictions.
