# Supplementary material for: Molecular Evidence of Human T‑Cell Leukemia Virus Type 1 Transmission by Needlestick Injury in Healthcare Workers
Source: J Med Virol. 2026 Sep 10;98(9):e71143. doi: 10.1002/jmv.71143 (PMC13560615; doi:10.1002/jmv.71143)
Supplement: Supplementary file 1 [file JMV-98-e71143-s002.docx]

Supplemental Tables

Supplemental Table S1 Genome-specific primer probe for HTLV-1 proviral load dPCR.

| Target | Primer-probe name | Sequence | | Final concentration (nM) | Dye |
| --- | --- | --- | --- | --- | --- |
| HTLV-1 | 184-MGB | Forward | CTAACAGCCTGGCAAAACG | 800 |  |
|  |  | Reverse | CAGGGCCCGGAAATCATAG | 800 |  |
|  |  | Probe | CCAGGCCTTATTTGGA | 200 | **5'FAM**/3'BHQ1 |
| Cellular gene | RPP30 | Forward | AGATTTGGACCTGCGAGCG | 800 |  |
|  |  | Reverse | GAGCGGCTGTCTCCACAAGT | 800 |  |
|  |  | Probe | TTCTGACCTGAAGGCTCTGCGCG | 200 | **5'VIC**/3'MGB |

HTLV-1, human T-cell lymphotropic virus type 1; dPCR, digital polymerase chain reaction

These primers have been previously reported in references S1 and S2 [1, 2]

Supplemental Table S2: Nested genomic long-PCR primers for the HTLV-1 full-length genome sequence

|  |  | **Name** | **Sequence** | **Name** | **Sequence** | **Size (bps)** |
| --- | --- | --- | --- | --- | --- | --- |
| **Fragment 1** | **1st PCR** | **215F** | **GCTCGCATCTCTCCTTCAC** | **071R** | **CGGTGACTGTGGTGGAGTAAGAT** |  |
|  | **2nd PCR** | **005F** | **CCTGACCCTGCTTGCTCAAC** | **066R** | **GGGCATGTAGCCAGACAAGTG** | **3016** |
| **Fragment 2** | **1st PCR** | **053F** | **GGAGGCAGGCCATATCGAA** | **173R** | **CCGAACATAGTCCCCCAGAGA** |  |
|  | **2nd PCR** | **56F-A 56F-G** | **TAGTCCCACCCTGTTCGAAATG TAGTCCCACCCTGTTCGAGATG** | **153R** | **AGGGATGACTCAGGGTTTATAAGAGA** | **4184** |
| **Fragment 3** | **1st PCR** | **122F-dg** | **CCTRGGGTGCCAATCATGGA** | **008R** | **GGCAGTCAGTCGTGAATGAAAG** |  |
|  | **2nd PCR** | **137F** | **CCCCACCTGACGTTACCATT** | **005R** | **TCAGTCGTGAATGAAAGGGAAAG** | **2962** |

PCR, polymerase chain reaction

These primers have been previously reported in reference S3 [3]

Supplemental Figure legends

Supplemental Figure S1: Clonal dynamics of HTLV‑1–infected cells in Case 1 at 5 and 10 months.

A. Paired pie charts showing the relative abundance of HTLV‑1 proviral integration sites in Case 1 at 5 (a) and 10 (b) months. The integration sites detected at both points are shown as grey slices. Integration sites detected only at 5 months are shown in orange slices, whereas integration sites newly detected at 10 months are shown in blue. HTLV-1, human T-cell lymphotropic virus type 1

Supplementary References

1.	Hiraga K, Tezuka K, Nagata K, Koh KR, Nakamura H, Sagara Y, et al. Development of a novel multiplex digital PCR-based method for the detection of HTLV-1 proviral deletion. Journal of virological methods. 2025;332:115071. Epub 20241121. doi: 10.1016/j.jviromet.2024.115071. PubMed PMID: 39577671.

2.	Lu X, Wang L, Sakthivel SK, Whitaker B, Murray J, Kamili S, et al. US CDC Real-Time Reverse Transcription PCR Panel for Detection of Severe Acute Respiratory Syndrome Coronavirus 2. Emerg Infect Dis. 2020;26(8):1654–65. Epub 20200515. doi: 10.3201/eid2608.201246. PubMed PMID: 32396505; PubMed Central PMCID: PMCPMC7392423.

3.	Kuramitsu M, Sekizuka T, Yamochi T, Firouzi S, Sato T, Umeki K, et al. Proviral Features of Human T Cell Leukemia Virus Type 1 in Carriers with Indeterminate Western Blot Analysis Results. Journal of clinical microbiology. 2017;55(9):2838–49. Epub 20170712. doi: 10.1128/JCM.00659-17. PubMed PMID: 28701419; PubMed Central PMCID: PMCPMC5648719.
